# Unveiling the Role of the Proton Gateway, Uncoupling Proteins (UCPs), in Cancer Cachexia

**DOI:** 10.3390/cancers15051407

**Published:** 2023-02-23

**Authors:** Mit Joshi, Bhoomika M. Patel

**Affiliations:** 1 Institute of Pharmacy, Nirma University, Ahmedabad 382481, India; 2 School of Medico-Legal Studies, National Forensic Sciences University, Sector 9, Gandhinagar 382007, India

**Keywords:** cancer cachexia, uncoupling protein, mitochondria, muscle atrophy, metabolism, wasting syndrome, adipose tissue wasting

## Abstract

**Simple Summary:**

Cancer cachexia is a wasting syndrome mainly driven by chronic inflammation and high energy expenditure. The hyperactivation of the catabolic pathway leads to higher energy utilization by the body. Uncoupling proteins are involved in uncoupling the electron transport chain and thereby halting the ATP production and releasing energy in the form of heat, which increases the body’s overall energy utilization. UCPs may play an important role during cancer cachexia. This review aims to highlight the role of UCPs in cancer and cancer cachexia and provide new knowledge to tackle this wasting syndrome.

**Abstract:**

Uncoupling proteins (UCPs) are identified as carriers of proton ions between the mitochondrial inner membrane and the mitochondrial matrix. ATP is mainly generated through oxidative phosphorylation in mitochondria. The proton gradient is generated across the inner mitochondrial membrane and the mitochondrial matrix, which facilitates a smooth transfer of electrons across ETC complexes. Until now, it was thought that the role of UCPs was to break the electron transport chain and thereby inhibit the synthesis of ATP. UCPs allow protons to pass from the inner mitochondrial membrane to the mitochondrial matrix and decrease the proton gradient across the membrane, which results in decreased ATP synthesis and increased production of heat by mitochondria. In recent years, the role of UCPs in other physiological processes has been deciphered. In this review, we first highlighted the different types of UCPs and their precise location across the body. Second, we summarized the role of UCPs in different diseases, mainly metabolic disorders such as obesity and diabetes, cardiovascular complications, cancer, wasting syndrome, neurodegenerative diseases, and kidney complications. Based on our findings, we conclude that UCPs play a major role in maintaining energy homeostasis, mitochondrial functions, ROS production, and apoptosis. Finally, our findings reveal that mitochondrial uncoupling by UCPs may treat many diseases, and extensive clinical studies are required to meet the unmet need of certain diseases.

## 1. Introduction

“Cachexia” is a Latin term meaning wasting or weakness of the body due to a prolonged disease condition. Cachexia is diagnosed in many chronic illnesses such as cancer, AIDS, chronic obstructive pulmonary disease (COPD), congestive heart failure (CHF), stroke, and chronic kidney conditions [1]. Cancer cachexia can be defined as irreversible weight loss, loss of appetite, and loss of skeletal muscle along with a reduction in fat depots [2]. In the last decade, cachexia has been considered a clinical condition in cancer patients that results in early mortality, resistance to chemotherapeutic treatment, and a substantial decrease in quality of life [3]. In 2011, Fearon et al. published an article focused on the classification and diagnosis of cachexia in different populations [4]. In recent years, much preclinical and clinical research has been conducted to identify the underlying mechanism of cachexia in cancer patients. The main culprits of cancer cachexia are systemic inflammation [5], host immune response [6], and host–tumor interaction [7]. Due to the activation of these pathways, there is an increase in catabolic cellular and molecular pathways increasing the energy expenditure of the whole body. The imbalance between catabolic and anabolic pathways leads to the wasting of skeletal muscle and adipose tissues [8]. To date, many interventions have been conducted to target the pathophysiology of cachexia, but they have all failed. Drugs such as cytokine inhibitors, thalidomide, NSAIDs, melatonin, anamorelin, corticosteroids, omega-3 fatty acids, and progesterone analogs showed much less evidence in the amelioration of cachectic conditions in cancer patients [9]. Nutritional supplements and exercise are the only possible choice to treat cachexia, although the results are controversial. To develop a new intervention, there is an urgent need to identify new targets in cancer cachexia pathophysiology.

As indicated in [10], UCPs are present in the inner mitochondrial membrane [11]. Mitochondria have a diverse role in biochemical processes such as the generation of adenosine triphosphate (ATP) through oxidative phosphorylation, involved in several steps of the citric acid cycle, urea cycle, and gluconeogenesis also take place in mitochondria. The outer membrane is permeable toward small molecules, whereas the inner membrane has selective permeability to generate an electronic gradient between the outer and inner membrane for the synthesis of ATP [12].

The main task of the mitochondrion is to regenerate the energy required for the normal functions of the cell and body. The cellular mechanism through which it facilitates the regeneration of energy in a form of ATP is known as the “electron transport chain” (ETC) or “cellular respiration” [13]. We will use the term “ETC” for the rest of the article. As the name suggests, the electron transport chain involves the transfer of electrons through the chain of different complexes ranging from complex I to complex IV. Reduced cofactors, such as nicotinamide adenine dinucleotide (NADH) and flavin adenine dinucleotide (FADH_2_), obtained from oxidized molecules via glycolysis and citric acid cycle, undergo a series of electron transfers. NADH yields three molecules of ATP, while FADH_2_, which enters into ETC at complex II, yields two molecules of ATP. The transfer of electrons through ETC controls the pumping of H+ ions from the mitochondrial matrix to the intermembrane space, thereby creating a proton gradient. The proton gradient generates energy, which is utilized by ATP synthase to phosphorylate ADP to generate ATP [13].

ETC is not accurate, and the energy liberated from the oxidation of molecules does not convert into ATP; instead, some energy is lost in a form of heat. H+ ions re-enter the matrix independent of ATP synthesis, and this is known as a proton leak [14]. There are two types of proton leak: inducible and non-inducible (basal). Non-inducible or basal proton leak is not regulated by a particular transporter, and the transfer of H+ ions across the membrane is not mediated by UCP. The transfer of H+ ions depends on the composition of fatty acyl present in the inner membrane and the presence of adenine nucleotide translocase. The inducible proton leak is highly controlled, with UCPs playing an important role in this process. The proton gradient created from proton transfer across the inner mitochondrial membrane acts in a supply-and-demand manner. The transfer of electrons through different complexes pumps the proton from the matrix to the inner membrane, while ATP synthase drives the proton from the inner membrane to the matrix to generate ATP [15].

The exact mechanism behind basal proton leak is not yet deciphered. Few studies have enlightened the role composition of inner membrane lipids, mainly fatty acyls. Although only one-third of protons leak through the lipid bilayer [16], and the majority of proton leak is facilitated by the abundance of adenine nucleotide translocase in the inner membrane, it should be noted that proton leaks are dependent upon the abundance of adenine nucleotide translocase (ANT) and not on their activity. Compared with inducible proton leak, the conductance of proton through ANT is considered negligible, as an abundance of ANT is considered very low in the inner membrane of mitochondria [17,18].

The inducible proton leak is mainly facilitated by uncoupling proteins [15]. Researchers are now curious to identify the exact role and pathophysiology of UCPs and inducible proton leaks in different diseases. Few studies have been carried out to identify the role of the different types of UCPs in the pathophysiology of different diseases. The role of UCPs in cancer and cachexia is discussed in the current article.

## 2. Uncoupling Protein 1

Brown adipose tissue (BAT) has been found to be activated in hibernating animals, in small rodents during cold exposure, and in infants at birth. The only role of BAT found in different physiological conditions is to induce thermogenesis. Brown adipose tissues are abundant with mitochondria and small lipid droplets [19]. UCP-1 is the first uncoupling protein found in brown adipose tissue, where it represents 10% of the mitochondrial content and is involved in the thermogenesis process via proton leak. There are two types of adipose tissue in which the expression of UCP-1 was predominantly found: brown adipose tissue and beige adipose tissue. The expression of UCP-1 in brown adipose tissue remains constant even at the basal level, while the expression of UCP-1 in beige adipose tissue depends on the phenotype change from white adipose tissue to beige adipose tissue, the activation of the sympathetic nervous system, and peroxisome proliferator-activated receptor-γ (PPAR-γ) agonists. However, both types of tissue show an equal level of activation and distribution when stimulated and result in the same level of thermogenic activity [20]. The activation of the sympathetic nervous system in response to overfeeding and cold conditions stimulates BAT, which further activates UCP-1 and initiates non-shivering thermogenesis. The sympathetic nervous system stimulates thermogenesis by activating the classic cAMP pathway, which increases the mitochondrial number and size, activates the transcription and translation of UCP-1 protein, and increases the flow of free fatty acids to the mitochondria for heat generation. Prolonged exposure to a cold environment results in the phenotype change from white adipose tissue to beige adipose tissue, which increases the thermogenic capacity of the body [21]. In the beginning, it was thought that sympathetic stimuli are the only way to stimulate non-shivering thermogenesis, although many other factors, molecules, and hormones are involved in the activation of UCP-1 and further thermogenesis. The activation of UCP-1 is highly dependent upon the environmental temperature.

The UCP-1 present in brown adipose tissue is considered a major mediator of thermogenesis under two major stimuli: prolonged cold exposure and overfeeding. Prolonged exposure to cold temperatures results in the activation of the sympathetic nervous system, which facilitates lipolysis in WAT. Free fatty acids are released through the lipolysis process utilized by BAT and beige adipocytes to generate heat by increasing resting energy expenditure. These phenomena raised interest among researchers in the treatment of obesity. Early research work was focused on identifying the role of UCP-1 in the regulation of thermogenesis. However, the preclinical data retrieved from different studies highlight the significant influence of ambient temperature.

UCP knockout mice with C57BL/6J background demonstrated resistance to diet-induced obesity at subthermoneutral temperature (20 °C). The possible mechanism behind the resistance was the alternate mechanism that maintains the body temperature. Thus, resistance was quickly reversed at a thermoneutral temperature (27 °C) [22]. A similar type of study also showed that at a thermoneutrality temperature (30 °C), the ablation of UCP-1 in mice resulted in obesity under the influence of a high-fat diet. The reason behind UCP-1 ablation resulting in obesity is that under thermoneutrality conditions, increased metabolism is not required to maintain the temperature, but under subthermoneutrality conditions, thermal stress is present, and the body utilizes either brown fat in the presence of UCP-1 or other mechanisms such as shivering thermogenesis in the absence of UCP-1, so the exact role of UCP-1 in BAT may be masked by other complementary mechanisms [23].

## 3. Uncoupling Protein 2

Having a close homology to UCP-1, the exact role of UCP-2 is still debatable. The distribution of UCP-2 had been found in adipose tissue, the central nervous system, the immune system, the kidney, and the brain. Based on its high distribution throughout the body, UCP-2 may have a role in several diseases such as obesity, diabetes, cardiovascular disease, neurodegenerative, and psychological disease [24]. Many research groups have published contrasting results regarding the role of UCP-2 in different diseases. The polymorphism of UCP-2 gene such as −866G>A (rs659366), Ala55Val (rs660339), −5331G>A, exon 8 deletion/deletion, and 45 bp insertion/deletion in 3′UTR results in obesity, type-2 diabetes, and neural tube defects [25,26].

## 4. Uncoupling Protein 3

Discovered in 1997, UCP-3 has been found in the skeletal muscle, the heart muscle, and adipose tissue. Initially, UCP-3 was thought to have a thermogenic effect, as it has a similar homology to UCP-1 [27]. However, unlike UCP-1, which has a life cycle of 30 h [28], UCP-3 has a very short half-life of 30 min, which makes it difficult for any molecular analysis [29,30]. A study using UCP-3 knockout (KO) mice revealed that UCP−/− 3 mice did not exhibit thermogenesis problems and did not show obesity. Increased levels of reactive oxygen species (ROS) were identified in UCP-3 KO mice, as well as in UCP-3 Tg mice. Although the role of UCP-3 as an antioxidant has remained controversial due to the lack of data regarding the correlation between UCP-3 and ROS production, UCP-3 is also thought to be involved in β-oxidation [27].

## 5. Uncoupling Proteins 4 and 5

Same as other UCP proteins, UCP-4 is thought to be involved in the thermogenesis process in brown adipose tissue, as UCP-4 has a similar homology to other UCPs [31]. Earlier studies have revealed that UCP-4 and UCP-5 are mainly present in the brain [32]. UCP-4 is mainly expressed in neurons, the hippocampus, the cortex, the substantia nigra, the striatum, and the cerebellum [32,33,34], while UCP-5 is expressed in the amygdala, the hippocampus, the mediodorsal and paraventricular thalamic nucleus, and the dorsomedial hypothalamic nucleus [35,36]. Both UCP-4 and UCP-5 are involved in the uncoupling of oxidative phosphorylation by providing a gateway to H+ ions and reducing oxidative stress and thereby protecting the mitochondria from an overload of oxidative stress [37]. UCP-4 is able to protect mitochondrial depolarization and decrease oxidative stress against MPP+-induced toxicity in SH-SY5Y cells, while another study showed the involvement of UCP-4 in maintaining calcium homeostasis and apoptosis in PC12 cells. UCP-5 is also involved in protecting SH-SY5Y cells from MPP+ and dopamine toxicity by preserving mitochondrial membrane potential and decreasing oxidative stress and ATP levels. These studies highlight the function of UCP-4 and UCP-5 in brain homeostasis and their possible role in neurodegenerative diseases.

A study evaluating the role of UCP-2 and UCP-3 in obesity found that the overexpression of UCP-2 and/or UCP-3 resulted in a decrease in fat mass with an increase in LDL cholesterol in mice, thus highlighting the role of UCPs in alleviating obesity [38]. Another study evaluating the role of UCP-1 and BAT in an obesity-resistant 129S mice strain found that the ablation of UCP-1 resulted in obesity even in obesity-resistant mice. That study also highlighted that the expression of UCP-1 increased, having a positive correlation with weight, and UCP-1 may counteract weight gain in mice fed with a high-fat diet and cafeteria diet [39]. Another study demonstrated that the gut microbiota was able to enhance the effect of curcumin by activating UCP-1-dependent thermogenesis, which prevents weight gain in diet-induced obese mice [40]. These studies revealed that the activation of UCPs in WAT and BAT leads to high energy expenditure followed by weight loss. The activation of UCPs in obese individuals plays an important role to halt weight gain but has a detrimental effect during cancer cachexia.

Several studies involved in identifying the role of UCPs in cardiovascular complications [41,42,43,44,45,46,47,48], neurodegenerative disorders [49,50,51], and kidney diseases [52,53,54,55] found that the activation of UCPs decreases oxidative stress, suppresses inflammation, and induces protective effects. However, in cancer and cancer cachexia, UCPs play a negative role and accelerate cachexia in cancer patients.

## 6. Role of UCPs in Cancer

Warburg et al. found that cancer cells use the glycolysis pathway for energy production even in an aerobic environment. The uniqueness of cancer cells is now known as the “Warburg effect” [56]. According to the phenomena, cancer cells utilize the glycolysis pathway due to defects in mitochondrial respiration. The Warburg effect is one of the important hallmarks of cancer and plays a central role in providing energy to fast-growing and differentiating cancer cells [57]. The study conducted by Negre-Salvayre et al. was one of the first studies to identify the role of UCPs in ROS generation. This study found that UCP-2 was able to decrease ROS production in the mitochondria [58]. Another study found that the formation of tumors in a colon carcinoma mice model lacking UCP-2 resulted in higher ROS production, proliferation, and NF-кB production, and decreased apoptosis. The study concluded that higher ROS levels may contribute to tumor progression in UCP-2-deficient mice. Nevertheless, no tumor invasion was detected, which questions the role of UCP-2 in the tumor microenvironment [59]. Subsequent studies revealed that the low expression or deletion of UCP-2 resulted in increased ROS production in the mitochondria. The activation of UCP-2 may play an important role in decreasing ROS production but has a detrimental effect on cancer because many studies have established that the overexpression of UCP-2 in cancer cells decreases ROS production in cancer cells and helps them to thrive [60].

An in vitro study on human colon cancer, using the HCT116 cell line, showed that the overexpression of UCP-2 protects the cells from apoptosis as well as oxidative stress, while the in vivo data showed resistance to anticancer drugs against HC-16-induced cancer in NCr *nu*/*nu* mice [61]. Another study also demonstrated that low UCP-2 levels in lung cancer cells can facilitate STAT3 activation and subsequent ROS generation by a chemotherapeutic agent. This study also showed the role of UCP-2 in cancer cell survival and drug resistance [62]. Another research group demonstrated that gemcitabine-induced UCP-2 mRNA expression, which in turn resists gemcitabine-induced damage to cancer cells, elucidates UCP-2-induced resistance [63]. The upregulation of UCP-3 changes the mitochondrial-induced oxidative stress during hypoxia/reoxygenation in vitro in partial H/R-resistant proximal convoluted tubule (PT) cells. As tumor hypoxic cells accelerate malignancy and drug resistance, targeting UCP-3 may help develop anticancer therapies [64]. Another study showed that UCP-2 expression in cancer cells determined the immunomodulatory function of the tumor microenvironment (TME) and had a direct effect on the survival of cancer cells. The activation of UCP-2 altered the cytokine signaling in an interferon-regulatory-factor-5-dependent manner. UCP-2 activated type-1 dendritic cells and CD8+ T cells and normalized TME. That study concluded that the induction of UCP-2, either through genetic modification or a pharmacological approach, resulted in UCP-2 making melanomas cells prone to programmed cell death via protein-1 blockade treatment and thus led to antitumor effects [65]. These studies found that the expression of UCP-2 is initially suppressed, which allows ROS production by cancer cells, and its overexpression in later stages leads to the inhibition of apoptosis and drug resistance. A clinical trial study revealed that HER2-positive breast cancer patients had overexpressed UCP-2 in tumor samples receiving the trastuzumab drug. Treatment with genipin, a UCP-2 inhibitor, significantly increased the antitumor effect of trastuzumab and increased the apoptosis of cancer cells. This study highlighted the role of UCP-2 as a potential target to overcome drug resistance in HER2-positive breast cancer [66]. Another study showed that mitochondrial uncoupling through the activation of UCPs protects CD133(+) colon cancer cells from ROS generation. This study suggested that the use of genipin with a ROS-inducing agent could be useful to eliminate stem-like colon cancer cells [67].

## 7. Pathophysiology of Cancer Cachexia

The pathophysiology of cancer cachexia mainly involves higher catabolic signaling compared with anabolic signaling, which leads to a higher energy expenditure of the body. The hypermetabolic state of the body results in the wasting of the skeletal muscle and adipose tissues. Proinflammatory cytokines and other mediators released by the tumor itself into the bloodstream are involved in skeletal muscle and adipose tissue wasting [5,68]. Although the host immune system is predominantly involved in releasing catabolic mediators in response to the tumor, another important factor is radiotherapy or chemotherapeutic drugs, which cause the activation of danger-associated molecular patterns, thus leading to the activation of cytokines and systemic inflammation [69].

Skeletal muscle wasting is mainly mediated through the autophagy–lysosomal pathway and the ubiquitin–proteasomal pathway [70]. Shreds of evidence have suggested that muscle-specific E3 ubiquitin ligases specifically present in muscle such as MuRF-1 and atrogin-1 are the main culprits of skeletal muscle wasting through activating the proteolysis pathway [71]. The activation of molecular pathways such as NF-кB, p38 MAPK, and STAT3 pathways leads to the overexpression of catabolic proteins such as MuRF-1, MAFBX, and atrogins, which further activates autophagy and the proteasomal pathway for muscle atrophy. The activation of autophagy and the proteasomal pathway mainly depends upon the type of cancer cells and the response of an immune system to it. Mitochondrial alterations are another important factor in the promotion of skeletal muscle wasting. Increased mitochondrial oxidative stress results in the degeneration of the mitochondrial network, and the overexpressed Fis1 gene leads to the autophagy and apoptosis of the skeletal muscle [72]. The role of the mitochondria in skeletal muscle wasting in cachectic conditions has caused the mitochondria to be a potential novel target in treating skeletal muscle wasting [73]. Systemic inflammation is another important mediator of skeletal muscle wasting [5]. Preclinical lines of evidence have found elevated levels of TNF-α and that the blockade of TNF-α with antibodies leads to the alleviation of muscle wasting [74]. TNF-α also contributes to the activation of other cytokines such as IL-1 and IL-6, which accelerate skeletal muscle wasting [75,76]. TNF-α and IL-1 further activate the classic NF-кB signaling pathway. The activation of the NF-κB pathway leads to the activation of the ubiquitin–proteasomal pathway through the overexpression of E3 ligase genes atrogin-1 and MuRF1 [77,78]. NF-κB also inhibits the Akt pathway, which leads to increased FOXO activity, further activating UPS- and ALP-related genes (LC3 and Bnip3) [79]. NF-κB also inhibits myogenesis-related genes such as MyoD, Myf5, and MRF4 and decreases myoblast differentiation [80]. The leukemia inhibitory factor (LIF) secreted by tumors belongs to the IL-6 family [81] and has been reported to induce muscle atrophy in animal models. The immunological inhibition of LIF resulted in reduced skeletal muscle loss, thus confirming LIF’s role in cancer cachexia [82]. LIF is also involved in lipolysis and lipid catabolism by acting on adipocytes as well as the hypothalamus [83]. Parathyroid-related protein (PTHrP)m, along with other tumor-derived factors, causes severe skeletal muscle loss by increasing the activity of atrophy-related genes [84].

Another important complication in cancer cachexia is the loss of adipose tissues [85]. Adipose tissues are mainly known to be involved in the storage of excessive fat. However, many other functions are reported in the last few decades. One of the important functions is the browning of white adipose tissues. Many studies have reported the browning of adipose tissue during prolonged cold exposure. The study conducted by Wagner et al. reported a phenotype change from white adipose tissue (WAT) to brown adipose tissue in cachectic patients [86], while another study conducted by Patsouris et al. observed a WAT-to-BAT phenotypic change in burn patients [87]. This study concluded that the WAT-to-BAT change occurs during a hypermetabolic state irrespective of pathological conditions. During cancer cachexia, the tumor and host-derived factors lead to a phenotypic change from WAT to BAT. The activation of BAT leads to more heat production by the mitochondria through upregulating UCPs and thereby increasing the total energy expenditure of the body [88].

**Figure 1:**
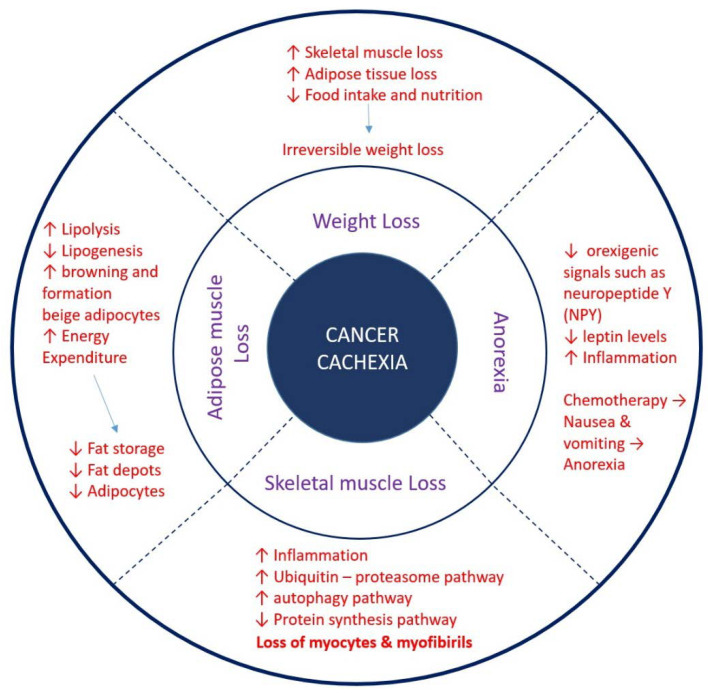
Features of cancer cachexia. Irreversible weight loss, anorexia, skeletal muscle loss, and adipose tissue loss are four cardinal features of cancer cachexia. Loss of adipocyte, skeletal muscle, and anorexia leads to irreversible weight loss in cachectic patients. Change in orexigenic signals, and imbalance in adipokine secretion results in anorexia. Side effects of chemotherapeutic drugs result in vomiting and nausea, leading to a decrease in food intake, while depression, a very common symptom among cancer patients, further promotes a decrease in food intake. Chronic inflammation due to the immune system and host–tumor interaction upregulates protein degradation cascade, mainly ubiquitin proteasomal and autophagy pathway, and downregulates protein synthesis pathway, leading to skeletal muscle loss primarily through acting on myocytes and myofibril formation. Inflammation and tumor-derived factors lead to increased lipolysis and decreased lipogenesis. High energy expenditure is initiated due to the activation of brown adipose tissue and phenotypic change from white to beige adipocytes. Complex cellular and molecular pathways lead to the initiation of cachexia in cancer patients.

During cachectic conditions, the tumor and host-derived factors, such as inflammatory cytokines, catecholamine, the leukotriene inhibitory factor (LIF) [89], and parathyroid-hormone-related protein (PTHrP) [84], lead to an increase in lipolysis in adipocytes, which results in increased liberation of free fatty acids in the circulation. These FFAs are taken up by beige or brown adipocytes and utilized to generate excessive heat [90,91] (Figure 1).

## 8. Role of UCPs in Cachexia

UCPs play a central role in inducing thermogenesis in humans, thereby increasing the energy expenditure of the body. One of the key features of cachexia is higher resting energy expenditure and thereby causing the atrophy of skeletal muscle and draining the adipose tissue. The involvement of UCPs in cancer cachexia was identified in different studies.

### 8.1. The Role of UCPs in Skeletal Muscle Wasting

As discussed above, the primary role of UCPs is to induce non-shivering thermogenesis. The upregulation of UCPs in the skeletal muscle during cancer progression is poorly understood. In one of the early studies, Sanchis et al. demonstrated that the rats bearing Yoshida Ah-130 ascites hematoma showed overexpression of UCP-2 and UCP-3 in the skeletal muscle after tumor induction. The study concluded that the overexpression of UCP-2 and UCP-3 in the skeletal muscle was due to the anorexia induced by the burden of the tumor. This was the first study highlighting the role of UCPs in cachectic conditions during cancer [92]. However, the same researchers further demonstrated contradictory results and showed that anorexia and high circulating FFAs were not directly linked with the activation of UCPs in an LLC tumor model [93]. Another study found decreased TCA cycle flux and ATP synthesis rate in Lewis lung carcinoma (LLC)-bearing mice compared with the normal control. The same study also found the overexpression of UCP-3, atrogin-1, FOXO3α, and PDK4 genes. The study reported that the overexpression of UCPs and mitochondrial uncoupling might be involved in the wasting of the skeletal muscle [94].

Mitochondrial alterations and impairment were observed in tumor-bearing cachectic mice. A previous study demonstrated that mitochondrial alterations led to muscle atrophy in LLC-bearing mice. In C26 tumor-bearing mice, the overexpression of the genes related to proteolysis, autophagy, and mitophagy was associated with decreased mitochondrial fusion proteins. Decreased levels of respiratory complexes I and II was observed in the muscle of tumor-bearing mice, which may be induced by increased levels of BNIP3-mitochondria (SDHa) colocalization. Previous studies also confirmed the downregulation of MFN2 and OPA1 in the atrophied muscle, which leads to altered mitochondrial function and muscle atrophy [95]. Another study showed a lower mitochondrial respiration rate and reduced mitochondrial coupling and found lower mitochondrial protein abundance in the soleus muscle of C26 tumor-bearing mice [96]. These studies showed that in cancer-bearing mice, the induction of the wasting of the skeletal muscle was due to a decrease in mitochondrial function, and the overexpression of UCPs was not the sole contributor to skeletal muscle loss. The same conclusion was drawn by another study conducted on Berlin–Druckrey IX rats with peritoneal carcinosis (PC), which showed that cancer cachexia was able to induce alteration in mitochondrial biogenesis and bioenergetics in skeletal muscles. The study found that alterations in mitochondrial activity were not due to the wasting or browning of AT but due to a decrease in the activity of complex IV, which resulted in decreased oxidative phosphorylation. This resulted in decreased ATP production in the mitochondria of the skeletal muscle. The overexpression of UCP-2 in the skeletal muscle was not associated with the wasting of the skeletal muscle. The overexpression of UCP-2 in quadriceps muscles did not change ROS production in PC rats compared with healthy pair-fed rats [97].

These studies showed that the overexpression of UCPs was found in the skeletal muscle during cancer progression. However, their direct involvement in the wasting process is still debatable, and other factors, such as mitochondrial dysfunction due to autophagy, mitophagy, decreased protein synthesis, and decreased activity of mitochondrial complexes, may be involved in skeletal muscle wasting during cancer.

### 8.2. The Role of UCPs in Adipose Tissue Wasting

Wagner et al. revealed that the browning of WAT starts at the early stage of cachexia and contributes to more energy expenditure and lipid mobilization. Increased energy expenditure is associated with increased mitochondrial activity and uncoupling mechanism in adipocytes. The thermogenic capabilities of interscapular BAT and subcutaneous WAT were found to be increased. The overexpression of UCPs and the browning of WAT contribute to increasing wasting in cancer conditions. Increased UCP-1 staining in the adipose tissue of cancer cachectic cancer patients showed that increased UCP activity was associated with more thermogenic activity in cancer cachexia and the inhibition of UCPs or the browning of adipose tissue may be beneficial to treat cancer cachexia [86]. A small clinical pilot study comparing cancer patients without weight loss vs. cancer patients with weight loss showed that patients with cancer-associated weight loss had decreased abdominal adipocytes, higher circulating IL-6, increased lipolysis through the overexpression of the ATGL gene, and the browning of adipose tissue through the UCP-activating gene PGC-1α. That study also found that Cidea may be involved in promoting WAT browning by inhibiting UCP-1 repression activity, indicating that the UCP-1 protein was detected in peritumoral white adipocytes in seven out of the eight patients having different types of the tumor but was not detected in ten weight-stable patients [98]. MAC16 tumor-bearing mice showed the overexpression of UCP-1 in BAT, which may be to counter the effect of the hypothermia generated by tumor-derived factors. BAT thermogenesis results in increased energy expenditure, leading to wasting syndrome. The overexpression of UCP-2 and UCP-3 in the skeletal muscle may result in decreased food intake and increased lipid mobilization through increased lipolysis from WAT [99]. A lipid-mobilizing factor was extracted and purified from the urine of cancer cachexia patients who had a weight loss of more than 10%. When repeated i.v. injections of LMF were given to NMRI mice, they showed significant weight loss in gonadal fat mass as well as interscapular BAT. The secretion of leptin from adipocytes was significantly reduced, and mRNA levels of UCP-1, UCP-2, and UCP-3 were significantly increased. The upregulation of UCP-1, UCP-2, and UCP-3 in BAT and UCP-2 in the skeletal muscle and liver suggested that UCPs were involved in utilizing an excess of fat to induce heat production and thereby increased catabolism and energy expenditure in cancer cachexia [100].

**Figure 2:**
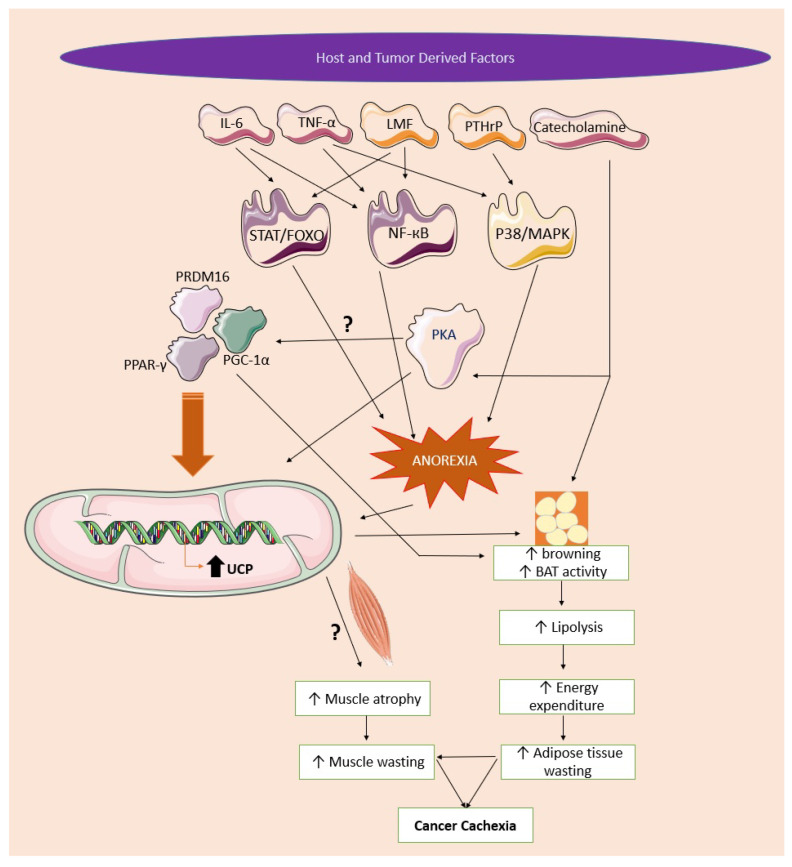
Molecular mechanism of UCP in cancer cachexia. Host and tumor-derived factors such as IL-6, TNF-α, catecholamines, PTHrP, and LMF cause activation of STAT/FOXO, NF-кB, and p38/MAPK, leading to activation of PKA, as well as PPAR-γ, PGC-1α, and PRDM16. Activation of these proteins leads to differentiation and phenotypic changes in adipocytes. This protein causes activation of UCPs in white, beige, and brown adipose tissues, causing increased lipolysis and energy expenditure. High energy expenditure causes loss of adipose tissue and skeletal muscle, ultimately leading to cancer cachexia. Activation of STAT/FOXO, NF-кB, and p38/MAPK is also involved in the pathophysiology of anorexia, which further causes activation of UCPs and results in skeletal muscle and adipose tissue wasting and cachexia. The upregulation of UCPs in skeletal muscle, and its role in skeletal muscle wasting in cancer cachexia, are still debatable, and further studies are required to decipher its role. IL-6: interleukin-6, TNF-α: tumor necrosis factor-α, LIF: leukotriene inhibitory factor, PTHrP: parathyroid hormone-related protein, STAT/FOXO: signal transducer and activator of transcription/class O forkhead transcription factors, NF-кB: nuclear factor кB, p38/MAPK: p38 mitogen-activated protein kinases, PKA: protein kinase A, PRDM16: PR domain containing 16, PPAR-γ: peroxisome proliferator-activated receptor gamma, PGC-1α: PPARG coactivator 1 alpha. ?—The exact role of UCP activation in skeletal musle during progression of cancer cachexia is still not known.

Many different interventions have been assessed to inhibit the browning of adipose tissue by inhibiting the overexpression of UCP-1 as well as the factors involved in the phenotype change from WAT to BAT. Luan Y et al. first reported that cachectic mice showed increased expression of p38 MAPK signaling, which activates thermogenesis in adipocytes by activating UCP-1 expression [101]. Another study reported that the p38 MAPK inhibitor SB203580 was able to block the activation of the MAPK pathway by inhibiting pancreatic tumor release exosomes in humans and 3T3-L1 adipocytes. P38α, a member of the p38 MAPK signaling pathway, is known to be involved in activating several proteins in lipid metabolism [102]. p38 MAPK inhibitors VCP979 and SB203580 were able to inhibit p38α and the β subunits of the p38 MAPK pathway, thereby decreasing the overexpression of UCPs and the browning of adipose tissue. These studies have shown the role of UCPs in activating the browning of WAT and increased energy expenditure due to it. The inhibition of UCPs or their upstream pathway may be beneficial to ameliorating the progression of cachexia in cancer [103]. A study conducted by Rohm et al. showed that decreased AMPK activity in the wasting of adipose tissue during cancer cachexia and targeting the WAT AMPK–Cidea interaction may prevent WAT browning in cachectic conditions. That study suggested that AMPK decrease occurs during the late stage of cachexia. It also questioned the stand-alone role of the overexpression of UCP-1 as a driving force of energy expenditure in cachexia. The study concluded that the inactivation of AMPK results in the browning and wasting of adipose tissue, and the stabilization of the AMPK activity in WAT prevents the browning and wasting of WAT [104]. Another study also supported the claims of the previous study and demonstrated that the extracts of *Arctium lappa* L. were able to lower the expression of UCP-1 by activating AMPK in WAT and BAT [105]. A study evaluating the role of free fatty acid (FFA) receptors FFA1 and FFA4 in cancer cachexia found that UCP-1 was not expressed in the interscapular WAT. Epididymis WAT showed UCP-1 expression in tumor-free mice, while it was downregulated in Lewis lung carcinoma-bearing mice regardless of treatment with GW9508 [106]. The same kind of result was obtained by another study on pancreatic cancer cachexia, which showed a reduction in UCP-1 expression in BAT and WAT with the progression of cachexia. The author concluded that the expression of UCP-1 increases in the early stage of cachexia, which may be due to sympathetic modulation [107] (Figure 2).

## 9. Conclusions

The current review sheds light on the role of UCPs and mitochondrial uncoupling. Very limited studies have been carried out to evaluate the exact role of UCPs in cancer-induced cachexia. UCPs are highly abundant in WAT and BAT and play important roles in non-shivering thermogenesis in the expanse of FFAs. Many studies have shown the upregulation of UCPs in adipose tissue. However, several studies highlighted the fact that even though UCPs were upregulated in WAT and BAT in cancer cachectic conditions, they were not exclusively involved in inducing cachexia. The upregulation of UCPs may be due to other factors involved during cachexia, and UCP activation just exaggerates the cachectic condition. In the context of the skeletal muscle, detailed studies are required to identify the role of UCPs in the context of cancer-associated cachexia. As discussed earlier, the upregulation of UCP-2 and UCP-3 was found to contribute to skeletal muscle loss during cancer cachexia, although some studies indicated that the upregulation of UCPs does not have any direct effect on skeletal muscle loss. More robust preclinical and clinical trials are required to unlock the mystery of UCPs in cancer cachexia. Based on the available studies, we can conclude that UCPs have two important roles. For diseases such as obesity, cardiovascular complications, and neurological complications, UCPs act as protagonists, while in cachexia and cancer, UCPs act as antagonists. The main role of UCPs is to maintain the proton flux across the mitochondrial membrane; nevertheless, UCPs impart many physiological changes in a particular disease condition. Different studies have revealed different responses from different UCPs, and most of the signaling remains complex and controversial and depends upon a vast number of other parameters. Thus far, UCP-1, UCP-2, and UCP-3 have been studied in a very limited way in cancer cachexia, and only their expression has been measured and identified, but no drug or molecule has been developed. The development of drugs acting on UCPs, along with targeting other molecules, could be a potential therapeutic option to halt or cure cachexia in cancer patients.
